# Dipsomania

**Published:** 1881-03

**Authors:** M. J. Halloran

**Affiliations:** Worcester, Mass.


### DIPSOMANIA.

By M. J. HALLORAN, M.D., Worcester, Mass.

Bruhl Cramer, a German physician, practicing at Moscow, described under the designation *Trunksucht*, an affection sometimes observed in habitual drunkards, where the appetite for liquor becomes so urgent that nothing will allay it. At present, under the designation dipsomania is understood a form of mental disease, having as a prominent symptom a morbid craving for drink, utterly beyond the power of the patient to control.

As Esquirol has pointed out, the patient may be noted for sobriety, even for his disgust for alcoholic stimulants, but at the moment of the access no respect for himself, for his relations or his position in society will prevent him from drinking. Often there are intervals of perfect sobriety. Esquirol records the case of a merchant who became dipsomaniac every winter; during the continuance of the attack nothing was spared, and he reduced himself almost to a beggar, but once the attack was finished, he return d again to business, and made good his losses. In the great proportion of cases these sober intervals become shorter, and sometimes the mad passion for drink seems to continue without lucid interval. (Fletcher—*British Medical Journal.*)

There is an acute form of dipsomania observed in persons predisposed to mental derangement on the occurrence of any great moral commotion, at the time of the change of life and during the puerperal state in women, or during the prodromal period of general paralysis or insanity in men.

In persons predisposed hereditarily to mental trouble, small quantities of alcohol soon induces a condition of veritable dipsomania; but it will generally be found that there exists in such individuals other evidence of a morbid mental condition, tendency to suicide, pyromania, etc.

"There is something wanting;" they all belong to the great class of degenerated beings (*etres degenerees*) on which Morel has so well written; they constitute the various classes of monomaniacs, a consideration which has led Skae to class dipsomania as one of the forms of moral insanity.

He remarks (Edinburg *Medical Journal*, 1858, p. 772:) "This craving for stimulants is only one of the symptoms of moral perversion which characterize the disease;" and again: "Of the other symptoms of moral perversion, the most common is the habit of lying; and accompanying this disregard for the truth; there are often other indications of moral perversion in the insane drunkard; such are extreme licentiousness, or a propensity to theft, or a delight in fomenting quarrels and creating mischief by leading other parties into trouble."

But there are other clinical facts which have not attracted the attention they merit.

I laid before M Magnan, of the St. Anne Asylum at Paris, the objections made by Bucknill to clinical observations of dipsomania in general and in particular to the one M. Magnan had, quoting from the elder Trelat, reproduced in his work on Alcoholism. It was the case of a lady whose mother and uncle had been subject to attacks of dipsomania. She led herself a very exemplary life, but from time to time entered on wild periods of drunkenness, during which friends, honor and family were forgotten. During these attacks she introduced all manner of nauseous substances into the liquor, in order to induce disgust of it, but without success. Dr. Bucknill contends that there is nothing to prove that she was not a common drunkard, given to periodical sprees, and rather takes to task M. Trelat for giving credence to her assertions of attempting to render the liquor nauseating.

M. Magnan replied to this objection: "The patient, previous to an attack of dipsomania, is just like the ordinary insane patient, distressed, plunged into profound melancholy, a prey to groundless fears." As M. Trelat observes (*de la Folie lucide*, p. 151:) "Drunkards drink when they find a favorable occasion; in dipsomania the patient drinks only at the moment of the access."

Some men attend well to business during the day, but drink heavily at night; others drink on public occasions or when driving a bargain; they drink, as M. Trelat says, when a favorable occasion offers.

How different the dipsomaniac: He may have every day liquor under his hands with no desire for it; at the moment of the access he will have recourse to every subterfuge to obtain liquor, and if deprived of it will swallow anything in the guise of a stimulant.

A young lady patient of Dr. Skae's drank creasote and vinegar, and even took tobacco, when deprived of everything else. And, unfortunately, such patients become dangerous while under the influence of liquor; a patient of Gall's became an incendiary only at such moments.

In the great majority of cases morbid hereditary antecedents exist; in thirty-two out of sixty-eight cases, according to Skae; such was also the case in several among the patients we observed at the St. Anne Asylum. We have found also that the patient was generally subject to other morbid impulses; one patient made three determined attempts at suicide; another was arrested twice in succession for petty thefts in public, giving more or less plausible reasons therefor.

These patients generally drink alone; they are often intensely ashamed of the vice; one patient used to immediately quit any house where she was discovered, leaving her furniture behind, and never returning to claim it.

The reasons given by these patients for their actions seem plausible, and they will make the most solemn promises of reform, which are invariably broken.

We consider, then, with Dr Skae, that dipsomania is one form of moral insanity, but that it is of difficult diagnosis; it has been too much the habit of late years to style inveterate drunkards "dipsomaniacs" Careful investigation should be instituted as to whether the ordinary actions of the patient give any indication of mental derangement.

As Morel remarks (*Etudes Cliniques*, p. 333:) "In such patients we recognize an unhappy organization tainted with morbid hereditary tendency, and destined to finish miserably in general paralysis or dementia."—*Medical and Surgical Reporter.*

